# Whole-genome sequencing of multidrug resistance *Salmonella* Typhi clinical strains isolated from Balochistan, Pakistan

**DOI:** 10.3389/fpubh.2023.1151805

**Published:** 2023-05-16

**Authors:** Sareen Fatima, Zaara Ishaq, Muhammad Irfan, Abdullah F. AlAsmari, Jahangir Khan Achakzai, Tahreem Zaheer, Amjad Ali, Ali Akbar

**Affiliations:** ^1^Department of Microbiology, University of Balochistan, Quetta, Balochistan, Pakistan; ^2^Atta-ur-Rahman School of Applied Biosciences (ASAB), National University of Sciences and Technology, (NUST), Islamabad, Pakistan; ^3^Jamil-ur-Rahman Center for Genome Research, International Center for Chemical and Biological Sciences (ICCBS), University of Karachi, Karachi, Pakistan; ^4^Department of Pharmacology and Toxicology, College of Pharmacy, King Saud University, Riyadh, Saudi Arabia; ^5^Disipline of Biochemistry, Department of Natural and Basic Sciences, University of Turbat Kech, Balochistan, Pakistan; ^6^Department of Biology, Indiana University Bloomington, Bloomington, IN, United States

**Keywords:** pathogenic bacteria, diseases, enteric infections, antibiotics resistance, superbugs

## Abstract

**Introduction:**

*Salmonella enterica* serovar Typhi (*S*. Typhi) is a major cause of morbidity and mortality in developing countries, contributing significantly to the global disease burden.

**Methods:**

In this study, *S*. Typhi strains were isolated from 100 patients exhibiting symptoms of typhoid fever at a tertiary care hospital in Pakistan. Antimicrobial testing of all isolates was performed to determine the sensitivity and resistance pattern. Three MDR strains, namely QS194, QS430, and QS468, were subjected to whole genome sequencing for genomic characterization.

**Results and Discussion:**

MLST analysis showed that QS194, belonged to ST19, which is commonly associated with *Salmonella enterica serovar typhimurium*. In contrast, QS430 and QS468, belonged to ST1, a sequence type frequently associated with *S*. Typhi. PlasmidFinder identified the presence of *IncFIB(S)* and *IncFII(S)* plasmids in QS194, while *IncQ1* was found in QS468. No plasmid was detected in QS430. CARD-based analysis showed that the strains were largely resistant to a variety of antibiotics and disinfecting agents/antiseptics, including fluoroquinolones, cephalosporins, monobactams, cephamycins, penams, phenicols, tetracyclines, rifamycins, aminoglycosides, etc. The *S*. Typhi strains possessed various virulence factors, such as *Vi antigen, Agf/Csg, Bcf, Fim, Pef,* etc. The sequencing data indicated that the strains had antibiotic resistance determinants and shared common virulence factors. Pangenome analysis of the selected *S*. Typhi strains identified 13,237 genes, with 3,611 being core genes, 2,093 shell genes, and 7,533 cloud genes. Genome-based typing and horizontal gene transfer analysis revealed that the strains had different evolutionary origins and may have adapted to distinct environments or host organisms. These findings provide important insights into the genetic characteristics of *S*. Typhi strains and their potential association with various ecological niches and host organisms.

## 1. Introduction

Typhoid fever has been found as a major cause of mortality and illness in developing countries (1) This indicated its potential contribution in the social and economic cost of the diseases globally (2). Typhoid fever is caused by the Gram-negative *Salmonella enterica* Serovar Typhi. Around 14 million cases of typhoid and paratyphoid fever were observed in 2017, with over 130,000 fatalities. Among others, only South Asia accounted for approximately 70% among these deaths, indicating the importance of the pathogen, contributing for large percentage of the global disease burden (3, 4). Annually, around 0.155 M fatalities were observed to be caused from non-typhoidal *Salmonella* serovars as well, causing diarrhea and other symptoms (5, 6).

The *Salmonella* Reference Service (SRS) of Public Health England (PHE) received an estimated 8,000 isolates each year from local and regional hospital labs (2).

Typically, *Salmonella* Typhi was shown to be host-specific, monophyletic, seldom undergone recombination events and displayed differences due to genomic degradation Multiple virulence encoding genes, either chromosomally or on the virulence-associated plasmid, have been linked to its pathogenicity. Bacterial adhesion to the intestinal epithelium and cellular invasion is mediated, in part, by fimbriae virulence genes such those producing long polar fimbriae (*lpf*). The *invA* gene was also shown to have a role in host identification and invasion of intestinal mucosal epithelial cells (7). Similarly, *msgA*, *pagC*, and *tolC*, were shown to contribute to intracellular or macrophage survival. Evidence suggested that the *spaN* gene contributes to *Salmonella*’s invasiveness by allowing the pathogen to invade non-phagocytic cells and kill off macrophages. The *cdtB* gene, which encodes the toxin, was also discovered to have a role in host identification and invasion (8). According to reports, the *spvC* gene, which is found mostly on the virulence plasmid, helps *Salmonella* proliferate and survive within the host cell. Additionally, systemic *Salmonella* infections were linked to this phenomenon (9).

Acquisition of one or more specific pathways is commonly linked to the development of multidrug resistance (MDR) in *Salmonella* Typhi. The already high prevalence rates of typhoid fever have been exacerbated by the emergence of *Salmonella* Typhi isolates resistant to drugs prescribed for treatment. Antibiotic-resistant forms of *Salmonella* Typhi are a serious threat to public health, according to the Centers for Disease Control and Prevention (10). Typhoid was traditionally treated with the first-line antibiotics chloramphenicol, ampicillin, and trimethoprim-sulfamethoxazole. The emergence of multidrug-resistant *Salmonella* Typhi, which cannot be treated with standard antibiotics, dates back to the 1980s (11). MDR *Salmonella* Typhi infections might thereafter be treated with fluoroquinolones and third-generation cephalosporin. It is crucial to identify the potential negative effects of antibiotics on virulence mechanisms like motility because patients infected with antibiotic-resistant *Salmonella* isolates were more likely to experience morbidity than those infected with susceptible isolates (12).

Preceding the advent of polymerase chain reaction (PCR), *Salmonella* isolates were classified into serovars based on the reaction of rabbit antisera with lipopolysaccharide (O antigen encoded by *rfb* genes) and flagellar antigens (phases 1 and 2 of H antigen encoded by *fliC* and *fljB*). The organism was separated into almost 2,600 serovars using this strategy, which relied on their phenotypic variance. While initially promising, this approach was ultimately discredited due to its high cost, high expert labor requirements, and inability to reveal genetic differences among serovars (2). In contrast, numerous types of typing have been reported to be used routinely as a key component of detection and investigation, such as Multilocus variable number of tandem repeat analysis (MLVA) (13). Pulsed-field gel electrophoresis, ribotyping, Repetitive extra genic palindromic sequence-based PCR (rep-PCR), and a combined PCR and sequencing-based approach that directly targets O- and H-antigen. However, the development of Whole Genome Sequencing (WGS) technology has improved the notion by providing a low-cost, high-throughput alternative to conventional typing for public health monitoring and epidemic identification (14).The method also allowed for the resolution of bacterial strains down to the single nucleotide level, which aided in tracing the origin of an infection (10) and in classifying isolates into more refined taxonomic clones (such as those specified by serotyping) (2).

## 2. Methodology

### 2.1. Study design

This is a cross sectional study where three *Salmonella* isolates including QS194, QS430, and QS468 were collected between August 2019 and August 2020 from Sandeman Provisional Hospital, Quetta Balochistan, Pakistan. These isolates were selected for whole genome sequencing on the basis of drug resistance.

### 2.2. Isolation of organism

The collected blood sample was cultured on *Salmonella*–*Shigella* Agar and MacConkey agars to cultivate bacteria by incubating them at 37°C for 24 h. Later, non-lactose fermenting colonies were confirmed through biochemical testing and antibiotic sensitivity.

### 2.3. Identification of drug resistance through antimicrobial susceptibility testing

The disk diffusion technique was used to determine antimicrobial sensitivity and resistance after isolating colonies and growing them on Mueller-Hinton agar (Oxoid) at 37°C for 24 h. Following antibiotic disks (Oxoid) were used with the given concentrations: ampicillin (10 μg), chloramphenicol (30 μg), streptomycin (10 μg), sulfonamide (300 μg), trimethoprim (5 μg), ciprofloxacin (5 μg), tetracycline (30 μg), gentamicin (10 μg), nalidixic acid (30 μg), Cefotaxime (5 μg), Mecillinam (10 μg) and Imipenem (10 μg). The criteria published by the Clinical and Laboratory Standards Institute (CLSI) was used for the interpretation of the results.

### 2.4. Molecular confirmation of *Salmonella* Typhi

#### 2.4.1. Oligonucleotide primers

Oligonucleotide primer sequence used in this study for the amplification of *invA and fliC* gene of *Salmonella* Typhi described earlier by Rahn et al. (15) and Song et al. (16) are shown in (Supplementary Table S1).

### 2.5. Whole genome sequencing

#### 2.5.1. Preparation of DNA library

DNA library preparation using high-quality bacterial DNA (50 ng) was accomplished by following the manufacturer’s instructions for the Nextera XT kit (Illumina, San Diego, CA, US). After fragmenting genomic DNA, it was ligated to adapters with individual indexes and then amplified using polymerase chain reaction (PCR). The PCR products were purified by using Agencourt Ampure beads. The concentration of the purified DNA was evaluated by DNA high sensitivity qubit kit using Fluorometer 2.0 Qubit. Three DNA libraries were combined into one with a representational concentration of one. Size distribution of the pooled library was estimated by 3% agarose gel electrophoresis. The library concentration was adjusted from ng to 4 nM and was denatured using 0.2 N NaOH. The denatured library was diluted to final concentration of 16 pM by using hybridization buffer (HT1).

#### 2.5.2. Genome sequencing and assembly and annotation

The library was subjected onto the MiSeq Illumina platform for paired-end high through put sequencing using 2 × 150 bp flowcell chemistry. With PE sequencing, both forward and reverse directions are sequenced on MiSeq Illumina. The total number of short sequenced reads 4,297,792 of QS194, 4,348,108 of QS430, and 4,179,946 of QS468 were generated.

FastQC package was used to assess the quality of the sequenced data and Trimmomatic v0.39 tool was employed to improve the quality of sequenced data by removing the adapter sequences. Adapter sequences, low quality bases, or such reads containing bases having size less than 36 were eliminated. After filtration, sequenced reads: 4270468 for QS194, 4.347290 for QS30 and 4,175,432 for QS468 were processed for *de novo* assembly using Unicycler (17) using SPAdes version 3.13.0 (18). The quality of the draft assembly was improved by pilon v1.23 tool (19). Filtered short sequenced reads of all the strains were aligned to the reference genome using BWA-MEM package to check the percentage of genome covered against the reference genome. The draft genome of all three *Salmonella* Typhi *strain*s; QS194, QS430, and QS468 were assembled into contigs. Quast package was used to assess the quality of assembled draft genome of each strain. The protein-coding genes and RNA-coding genes of the draft were located using FASTA sequences obtained from the fully assembled genome. The genomes of all three strains were assembled, and then annotated using RAST and the NCBI Prokaryotic Genome Annotation Pipeline (PGAP) v6.1.

#### 2.5.3. Genomic characterization of the isolates

In this study, we utilized the CGE services (^1^accessed on 28 March 2023) to conduct *in silico* epidemiological analysis of our isolates. We employed SeqSero 1.2 to predict the serotypes of *Salmonella* strains (20). While MLST 2.0 and PubMLST were utilized for multi locus sequence typing of the assembled genomes (21, 22). We used SPIFinder 2.0 to identify *Salmonella* Pathogenicity Islands, at default parameters (95% minimum identity threshold and 60% minimum coverage) (23). PlasmidFinder 2.1 was used to identify plasmids in the *Salmonella* isolates at default parameters (95% minimum identity threshold and 60% minimum coverage), the database selected was Enterobacteriales (24). Furthermore, we utilized ResFinder 4.1 to predict acquired antimicrobial resistance genes and chromosomal mutations with default parameters and the results were further confirmed by BLAST search at default parameters against Resistance Gene Identifier of Comprehensive Antimicrobial Resistance Database (CARD) (25, 26). CRISPRDetect 2.4 was employed at default parameters to predict and analyze CRISPR arrays in our isolates (27). We also identified and annotated virulence genes in our isolates using the Virulence Factor Database available at http://www.mgc.ac.cn/VFs/ accessed on 10 October 2022 (28).

#### 2.5.4. Evolutionary relationships inferred from pangenomes

One hundred fifty-six genome sequences of *Salmonella* Typhi and Typhimurium (95 complete genome sequences from different geographical locations and 61 WGS sequences of Pakistani strains) were downloaded from PATRIC (^2^accessed on 29 March 2023). Metadata regarding these genomes including strain name, BioSample, BioProject, accession numbers, GC% sequence type, isolate type and geographical location etc. is in Supplementary Table S2. For pangenome analysis, total 159 strains were used (95 complete genomes +61 Pakistani strains + Our 03 strains) which were then annotated using Prokka at default parameters (29). Then these annotated genomes were submitted to PanRV for pangenome estimation employing Roary, the core-genome SNPs based phylogenetic analysis was done and the tree was visualized and edited by iTOLv5 webserver (available at^3^ and accessed on 30 March 2023) (30–32).

## 3. Results

### 3.1. Isolation of bacterial strains

The culture media showed non-lactose fermenting colorless colonies, suggestive of the presence of *Salmonella*. Following observation was done for the confirmation of *Salmonella* species through biochemical testing (Supplementary Table S3).

### 3.2. Antimicrobial susceptibility testing

The antimicrobial susceptibility test for *Salmonella* Typhi has been performed on Mueller Hinton agar plate by the disk diffusion method. Result interpretation was conducted by following the Clinical and Laboratory Standards Institute (CLSI) guidelines (Supplementary Table S4).

### 3.3. Molecular confirmation by PCR

The presence of *Salmonella* Typhi was confirmed by Agarose Gel Electrophoresis. 2% Agarose Gel was used, and for confirmation *invA and fliC* genes were used which were 284 bp and 495 bp in size, respectively. Positive and negative controls were use (M1/M2 DNA marker) which were 100 bp apart from each other (Supplementary Figure S1).

### 3.4. Whole genome sequencing

#### 3.4.1. Genome characteristics

Paired end short sequenced data 1,055 MB, 1065 MB, and 1,028 MB of QS194, QS430, and QS468, respectively, was generated from the MiSeq Illumina. After removing the poor quality data using trimmomatic the obtained good quality sequenced reads, i.e., 4,270,468, 4,347,290, and 4,175,432 for QS194, QS30 and QS468, respectively, were *de novo* assembled by Unicycler followed by fine polishing using pilon package. The obtained high quality short sequenced reads data was mapped against the reference genome and found that 98.34% genome size of QS194 was covered against the reference genome followed by 89.4% QS430 and 87.48% for QS468. The entire genome was not assembled as a result of reference mapping. The sequenced data of all three strains QS194, QS430 and QS468 was assembled into draft genome of 4.59 MB, 4.48 MB and 4.38 MB, respectively, [Supplementary Figures S3a-c]. Annotation of these strains were performed by Prokaryotic Genome Annotation Pipeline (PGAP). The GC content of the sequenced strain was found on average of 52.39%. Table 1 summarizes the genome characteristics of three strains namely QS194, QS430 and QS468.

**Table 1:** Summary of genomic characteristics of three strains.

	QS194	QS430	QS468
Total Size	4.59 MB	4.48 MB	4.38 MB
Contigs	161	148	218
GC %	52.36	52.32	52.49
N50	45,757	51,613	33,113
tRNA	63	56	58
rRNA	6	3	3
Bioproject	PRJNA822699	PRJNA822699	PRJNA822699
Biosample	SAMN27259108	SAMN27362044	SAMN27512751
Accession numbers	JALIDY000000000	JAMKEL000000000	JALKCA000000000
Sequencing depth	99.96x	99.82x	86.66x
Coverage	98.34%	89.4%	87.48%
CDS	4,461	4,170	4,120
serovar	Typhimurium	Typhi	Typhi
Serotype	O antigen prediction: Not detected H1 antigen prediction (fliC): Present (y) H2 antigen prediction (fljB): Present for both types 1 and 2 (1,2) Predicted subspecies: I Predicted antigenic profile: -:y:1,2 Predicted serotype: I -:y:1,2	O antigen prediction: Not detected H1 antigen prediction (fliC): Present (d) H2 antigen prediction (fljB): Not detected Predicted subspecies: I Predicted antigenic profile: -:d:- Predicted serotype: I -:d:-	Predicted antigenic profile: -:d:- Predicted serotype(s): Not provided
MLST	ST19	ST1	ST1
Plasmids	IncFIB(S) IncFII(S)	No plasmid found	IncQ1
AMR genes	*mdsB, mdsA, golS, sdiA, Escherichia coli marR mutant conferring antibiotic resistance, Escherichia coli soxS with mutation conferring antibiotic resistance, Escherichia coli soxR with mutation conferring antibiotic resistance, Escherichia coli GlpT with mutation conferring resistance to fosfomycin, Escherichia coli UhpT with mutation conferring resistance to fosfomycin, Escherichia coli EF-Tu mutants conferring resistance to Pulvomycin, Haemophilus influenzae PBP3 conferring resistance to beta-lactam antibiotics, Klebsiella pneumoniae KpnE, Klebsiella pneumoniae KpnF, bacA, kdpE, CRP, PmrF, rsmA, emrR, emrB, AAC(6′)-Iaa, marA, msbA, vanG, acrB, Escherichia coli acrA, Escherichia coli mdfA, baeR, leuO* and *H-NS*	*sul1, qacEdelta1, catI, CTX-M-15, Escherichia coli soxS with mutation conferring antibiotic resistance, Escherichia coli soxR with mutation conferring antibiotic resistance, Escherichia coli marR mutant conferring antibiotic resistance, Haemophilus influenzae PBP3 conferring resistance to beta-lactam antibiotics, Escherichia coli UhpT with mutation conferring resistance to fosfomycin, Escherichia coli GlpT with mutation conferring resistance to fosfomycin, Salmonella enterica gyrA conferring resistance to fluoroquinolones, Escherichia coli mdfA, MdtK, H-NS, leuO, Escherichia coli acrA, CRP, AAC(6′)-Iy, Klebsiella pneumoniae KpnF, Klebsiella pneumoniae KpnE, emrB, emrR, msbA, rsmA, marA, sdiA, PmrF, bacA, vanG, baeR* and *kdpE*	*TEM-1, Escherichia coli marR mutant conferring antibiotic resistance, Escherichia coli soxR with mutation conferring antibiotic resistance, Escherichia coli soxS with mutation conferring antibiotic resistance, Escherichia coli UhpT with mutation conferring resistance to fosfomycin, Haemophilus influenzae PBP3 conferring resistance to beta-lactam antibiotics, Salmonella enterica gyrA conferring resistance to fluoroquinolones, Escherichia coli GlpT with mutation conferring resistance to fosfomycin, Escherichia coli EF-Tu mutants conferring resistance to Pulvomycin, marA, MdtK, Escherichia coli acrA, H-NS, msbA, baeR, bacA, emrR, emrB, rsmA, Klebsiella pneumoniae KpnE, Klebsiella pneumoniae KpnF, Escherichia coli mdfA, sdiA, leuO, sul2, APH(6)-Id, kdpE, PmrF, vanG* and *CRP*
Virulence factors	*Agf/Csg, Bcf, Fim, Lpf, Pef, Saf, Stb, Stc, Std, Stf, Sth, Sti, Stj, Mig-14, Mig-5, Mg2+* transport, *MisL, RatB, ShdA, SinH, PhoPQ, TTSS* (SPI-1 encode), *TTSS* (SPI-2 encode), TTSS effectors translocated via both systems, *TTSS-1* translocated effectors, *TTSS-2* translocated effectors, *Rck* and *SpvB*	*Vi* antigen*, Agf/Csg, Bcf, Fim, LpfC, Saf, Sta, Stb, Stc, Stc, Std, Ste, Stg, Sth, Tcf, Mig-14, Mg2+* transport, *MisL, RatB, ShdA, SinH, PhoPQ, TTSS* (SPI-1 encode), *TTSS* (SPI-2 encode), *TTSS* effectors translocated via both systems, *TTSS-1* translocated effectors, *TTSS-2* translocated effectors, Typhoid toxin	*Vi* antigen, *Agf/Csg, Bcf, Fim, LpfC, Saf, StaC, Stb, Stc, Stc, Std, Ste, Stg, Sth, TcfC, Mig-14, Mg2+* transport, *MisL, RatB, ShdA, SinH, PhoPQ, TTSS* (SPI-1 encode), *TTSS* (SPI-2 encode), *TTSS* effectors translocated via both systems, *TTSS-1* translocated effectors, *TTSS-2* translocated effectors, Typhoid toxin
SPI	C63PI, CS54_island, SPI-1, SPI-2, SPI-3, SPI-5, SPI-9, SPI-13 and SPI-14	SPI-1, SPI-2, SPI-3, SPI-5 and SPI-9	SPI-1, SPI-2, SPI-3, SPI-5 SPI-7 and SPI-9
CRISPR arrays	Array 1 (8747–10,224) Array family: I-E Array 1 (8882–7,328) Array family: I-E	Array 1 (61499–61,084) Array family: I-E	Array 1 (52595–52,173) Array family: I-E

#### 3.4.2. Genes associated with antibiotic resistance and virulence

The strains have been found to share several antibiotic resistance determinants including *sdiA*, *golS*, *mdsA*, *mdsB*, *H-NS*, *baeR*, *vanG*, *acrB*, *acrA* from *E. coli kdpE*, *mdfA* from *E. coli*, *msbA*, *KpnE* and *KpnF* from *K. pneumonia*, *emrR*, *emrB*, *rsmA* and *CRP*. These determinants provide resistance by antibiotic efflux, target alteration, antibiotic inactivation and reduced membrane permeability. Furthermore, mutations in genes such as *PmrF*, *bacA*, *PBP3* from *H. influenzae*, *GlpT* mutant from *E. coli*, *EF-Tu* mutants from *E. coli*, and *UhpT* from *E. coli* also confer resistance by target alteration and reduced membrane permeability. Mutations in *AcrAB-TolC* and *soxR* from *E. coli* result in resistance by target alteration and efflux, while mutations in *soxS* from *E. coli* provide resistance by target alteration, efflux, and reduced permeability. Additionally, QS430 has been found to possess these antibiotic determinants *CTX-M-15, catI, qacEdelta1*, *sul1 and Salmonella enterica gyrA* conferring antibiotic resistance by antibiotic efflux, target alteration and antibiotic inactivation. On the other hand, strain QS468 has found to possess *TEM-1* conferring resistance to beta lactamases by antibiotic inactivation. All these results are summarized in Table 1 above and have been visualized in Supplementary Figures S2–S4, respectively. Although the strains exhibited some common virulence factors, there were notable differences among them (Table 1; Supplementary Figures S2–S4). Specifically, the typhoid toxin genes *cdtB* and *pltA* were present in QS430 and QS468 but absent in QS194, indicating that QS194 is distinct from the other strains in this regard.

#### 3.4.3. Genome based typing and horizontal gene transfer

In our case, the MLST analysis revealed that QS194 belongs to ST19, a sequence type that is commonly associated with *Salmonella enterica* serovar Typhimurium. This result suggests that QS194 is likely to be closely related to other strains of *S*. Typhimurium with the same sequence type. This could indicate that QS194 may have originated from the same geographical region or has a similar ecological niche as other *S*. Typhimurium strains that share this sequence type (Table 1).

On the other hand, QS430 and QS468 were found to have ST1, which is a different sequence type from QS194. This suggests that these strains are likely to have a different evolutionary origin and may have adapted to different environments or host organisms. It is also worth noting that ST1 is a common sequence type associated with *Salmonella enterica* serovar Typhi, which causes typhoid fever in humans. The presence of typhoid toxin genes *cdtB* and *pltA* in QS430 and QS468 further supports their potential association with *S*. Typhi (Table 1).

PlasmidFinder identified two plasmids in QS194 strain with 100% similarity with *Salmonella enterica* subsp. *enterica* serovar Typhimurium strain D23580 plasmid *pSLT-BT* and *Salmonella enterica* subsp. *enterica* serovar Paratyphi C strain RKS4594 plasmid *pSPCV* (Table 1). It identified one plasmid in QS468 strain with 100% identity with *Escherichia coli* plasmid *RSF1010.* No plasmid was detected in QS430 strain (Table 1).

SPIFinder identified that our isolates share 5 *Salmonella* pathogenicity islands, SPI-1, SPI-2, SPI-3, SPI-5 and SPI-9. SPI-1 and SPI-2 are the most well-studied SPIs and are critical for *Salmonella*’s ability to invade and replicate within host cells. SPI-3 contains genes that enable *Salmonella* to evade the host immune response, while SPI-5 is involved in the transport and utilization of nutrients within host cells. Additionally, SPI-13, SPI-14, C63PI and CS54_islands were detected in QS194. SPI-9, SPI-13, and SPI-14 are involved in the transport and metabolism of specific nutrients that are important for *Salmonella* survival within the host. C63PI (Chromosome 63 Pathogenicity Island) contains several genes that are associated with virulence, including genes encoding type III secretion system (T3SS) effectors and regulatory proteins. CS54_island (Chromosome segment 54 island) is a pathogenicity island found in some strains of *Salmonella* and contains several genes that are associated with virulence, antibiotic resistance, and fitness, including genes encoding efflux pumps, iron acquisition systems, and proteins involved in biofilm formation. Additionally, SPI-7 was detected in QS468 which is a relatively large genomic region that contains several genes that have been implicated in the virulence of *Salmonella*.

CRISPRDetect identified two CRISPR arrays in QS164. The first array, located at positions 8,747–10,224 in the genome, belongs to the I-E family of CRISPR arrays. This suggests that QS164 possesses the genetic machinery necessary for a type I CRISPR-Cas system, which can be used by the bacterium to defend against foreign DNA elements such as bacteriophages and plasmids.

The second array, located at positions 8,882–7,328 in the genome, also belongs to the I-E family of CRISPR arrays. The presence of two CRISPR arrays in the QS164 genome suggests that this strain has a robust defense system against foreign DNA elements, which may contribute to its virulence and survival in different environments. While in QS430 and QS468 one array per each genome had been detected (Table 1).

#### 3.4.4. Comparative pangenomics and phylogenetic inference

The analyzed *Salmonella* strains exhibit an open pangenome which consists of total 13,237 genes, of which 3,611 (27.28%) are part of core genes, 2093 (15.8%) are shell and 7,533 (56.9%) are cloud genes. The statistics confirm the diverse nature of these strains with a large proportion of cloud genes, indicating a high degree of genomic plasticity.

**Figure 1:**
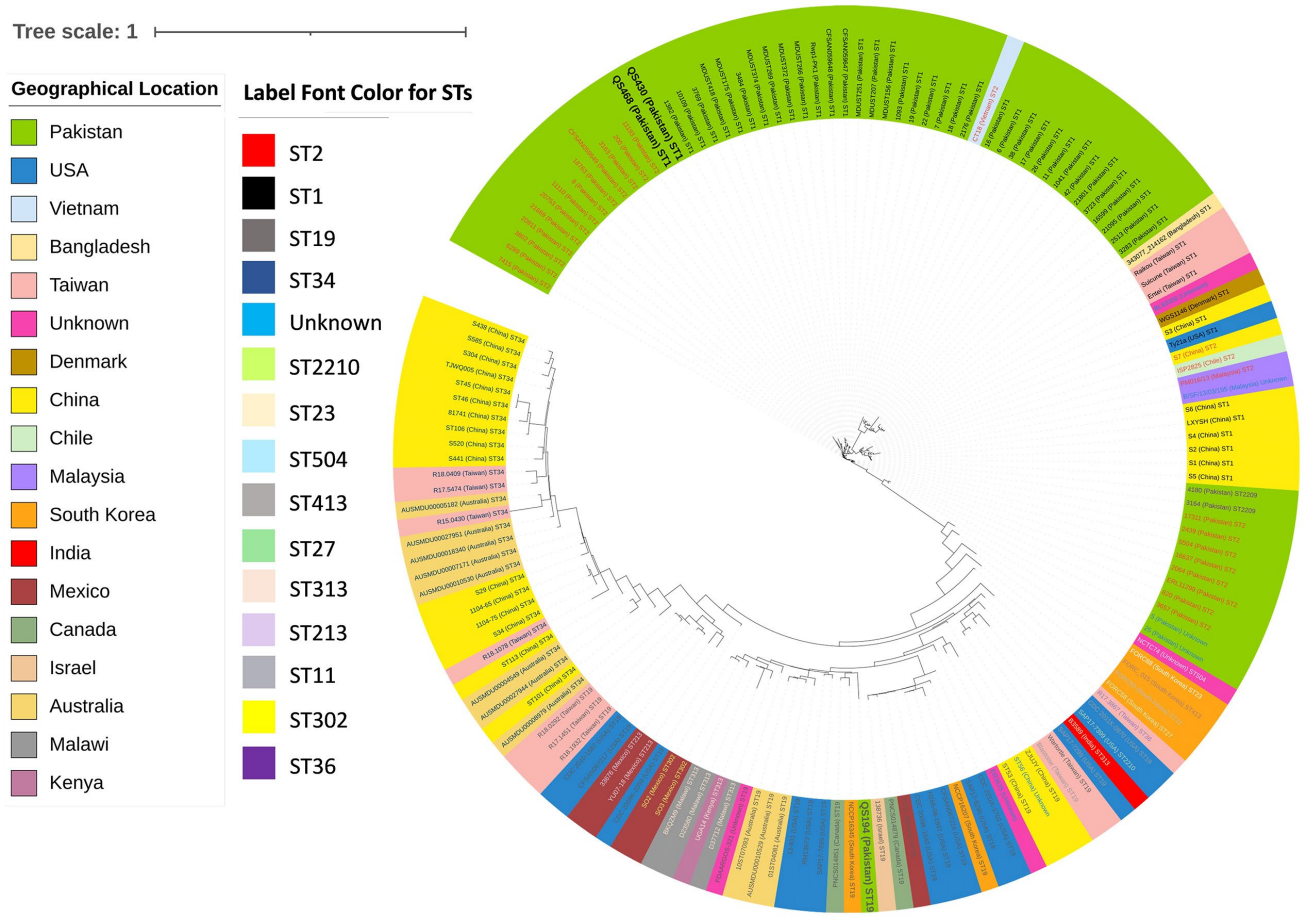
Core-genome SNPs based phylogenetic analysis of 159 (test strains *n* = 3, local strains *n* = 61, and global strains *n* = 95) publicly available *Salmonella* Typhi and Typhimurium strains showing diversity among local and global strains. Color key indicating geographical location and label font color for STs is provided along with.

The core-genome SNP based phylogenetics analysis revealed that strain QS430 and QS468 belong to the prevalent ST1 sequence type (both globally and locally) by sharing same clade with other local strains 1,382, 10,109, 3,679, MDUST418, MDUST175, 3,484, MDUST374, MDUST269, MDUST372, MDUST266, Rwp1-Pk, CFSAN059648, CFSAN059647, MDUST257, MDUST201, MDUST156, 1,093, 19, 22, 7, 18 and 2,176 with same ST. While QS194 clustered with other ST19 strains reported from worldwide and was found to be most closely related to NCCP16345 strain from South Africa and PNCS014851 strain from Canada (Figure 1). ST19 was found to be second most prevalent sequence type globally after ST34. These results highlight the importance of studying the diversity and evolution of *Salmonella* strains to better understand their global epidemiology and pathogenic potential.

Figure 1 indicates circularized core-genome SNP tree of 159 *Salmonella* strains showing same clade of QS430 and QS468 whereas QS194 belongs to the different clade. The most dominant sequence type was found to be ST1, including QS430 and QS468. QS164 belonged to ST19. Color key indicating STs and geographical location is provided alongside the circular tree.

## 4. Discussion

Typhoid fever has been recognized as a great health concern throughout the world, affecting the major economy of many developing and developed countries. Greater disease burden from typhoid and paratyphoid fever has been recorded particularly in low-income areas of Asia, Southeast Asia, and Sub-Saharan Africa in 2015 (33). This accounted for more than 15 million cases globally, only in 2015. However, the recent research stated that this number has been raised to more than 20 million cases annually around the globe. As *Salmonella* species have been recognized as the potential contributor in the emergence of the disease, there drug-resistant strains could increase its prevalence in high-risk countries (11, 34).

Antibiotic resistance is a significant public health threat, and *Salmonella* is a leading cause of antibiotic-resistant bacterial infections in humans. Several previous studies have reported the presence of antibiotic resistance determinants in *Salmonella* strains isolated from human hosts. A study by Randall et al. identified the presence of genes conferring resistance to multiple antibiotics, including tetracycline, sulfonamides, and beta-lactams, in *Salmonella* strains isolated from human patients in humans and animals (35). Similarly, a study by Xia et al. found that *Salmonella* strains isolated from human patients in China exhibited resistance to multiple antibiotics, including tetracycline, ampicillin, and ciprofloxacin, through the acquisition of antibiotic resistance genes (36). These studies highlight the urgent need for effective antibiotic stewardship programs and the development of new treatment strategies to combat antibiotic-resistant *Salmonella* infections in human hosts.

The findings of our study are consistent with previous studies that have characterized the genomic diversity of *Salmonella* isolates. A study by Zhou et al. analyzed the genomes of 1,500 *Salmonella* isolates from around the world and found that there is a high degree of genomic diversity among different serotypes and sequence types. In particular, they observed that certain sequence types, such as ST19 and ST34, are associated with multiple serotypes and are prevalent globally (37). This is consistent with our finding that QS194 belongs to ST19 and is closely related to other *S*. Typhimurium strains with the same sequence type.

Our study identified several antibiotic resistance determinants in the three *Salmonella* strains, including genes conferring resistance to beta-lactams, aminoglycosides, quinolones, and tetracyclines. Interestingly, QS430 and QS468 were found to possess the typhoid toxin genes *cdtB* and *pltA*, which are commonly associated with *S*. Typhi, the causative agent of typhoid fever. The presence of these genes in *Salmonella* strains has been previously reported and is a cause for concern as it suggests a potential transfer of these genes between different *Salmonella* serovars (38, 39).

Our study also identified mutations in several genes that confer antibiotic resistance by target alteration and reduced membrane permeability. This is in line with previous studies that have reported the role of mutations in conferring antibiotic resistance in *Salmonella* strains (40, 41).

Our observation that QS430 and QS468 have ST1, a sequence type commonly associated with *S*. Typhi, is also consistent with previous studies that have identified ST1 as a prevalent sequence type among *S*. Typhi isolates (42). Moreover, the presence of typhoid toxin genes in QS430 and QS468 further supports the potential association of these strains with *S*. Typhi.

Our analysis of plasmids and pathogenicity islands in the *Salmonella* isolates is also consistent with previous studies that have characterized the genomic features of *Salmonella* strains. A study by Ahmer et al. analyzed the plasmid content of 92 *Salmonella* isolates and identified a wide variety of plasmids with different sizes and functions (43). Similarly, a study by Langridge et al. (44) characterized the diversity of pathogenicity islands in *Salmonella* and identified a total of 26 different pathogenicity islands in the genomes of 90 *Salmonella* isolates (44).

The findings of the comparative pangenomics and phylogenetic inference analysis of the *Salmonella* strains are consistent with previous studies that have reported the high degree of genomic plasticity and diverse nature of these bacteria. A study by Leekitcharoenphon et al. found that the pangenome of *Salmonella enterica* consists of approximately 18,000 genes, with a large proportion of accessory genes that contribute to the variability and adaptability of the species (45). Similarly, a study by Ashton et al. identified a high degree of genomic diversity among *Salmonella* strains, with a large proportion of variable genes and a core genome that varies in size depending on the clade or serotype being analyzed (46).

The identification of ST1 and ST19 as prevalent sequence types in the *Salmonella* strains analyzed in the present study is consistent with global trends reported in previous studies. A study by Díaz-Torres et al. reported that ST1 and ST19 are among the most prevalent sequence types of *Salmonella enterica* worldwide (47). Similarly, a study by Wong et al. found that ST1, ST19, and ST34 are the most common sequence types of *Salmonella enterica* in Southeast Asia (48).

The identification of the relatedness between QS194 and ST19 strains reported from worldwide, such as NCCP16345 strain from South Africa and PNCS014851 strain from Canada, highlights the global distribution of *Salmonella* strains and their potential for causing outbreaks on a global scale. This finding is consistent with previous studies that have reported the global distribution of *Salmonella* strains and the importance of monitoring their diversity and evolution to better understand their epidemiology and pathogenic potential (45, 47, 48).

The correct surveillance and monitoring of control programs rely on the availability of rapid, simple, cost-effective, and improved diagnostics for pathogens identification. Finding biomarkers in typhoid carrier strains that need in-depth experimental examination is a difficult task, but whole genome sequencing has been a game-changer in this regard. There is currently no animal model that accurately represents human illness, and the enormous expenses of conducting experiments make this method difficult. Research into the typhoid carrier-state mechanism has allegedly started, but this area remains mostly unexplored. Consequently, there is still a need to incorporate significant efforts to solve this puzzling topic.

## 5. Conclusion

The study concluded that *Salmonella* Typhi has very diverse nature of strains among which certain genes were primarily involved in disease and antibiotic resistance, whereas others were involved in metabolic processes. Due to the ability to generate invasive infections and spread antimicrobial resistance (AMR) genes, *Salmonella* Typhi remains a formidable therapeutic challenge. There are no newer antityphoid medications in development, thus it is critical to examine full genomes to learn about their traits and look for new diagnostic targets.

## Data availability statement

The datasets presented in this study can be found in online repositories. The names of the repository/repositories and accession number(s) can be found at: https://www.ncbi.nlm.nih.gov/, JALKCA000000000; https://www.ncbi.nlm.nih.gov/, JAMKEL000000000; https://www.ncbi.nlm.nih.gov/, JALIDY000000000.

## Ethics statement

The study was approved by the Departmental Committee and Advanced Studies and Research Board with reference No. GSO/74, 01/2020. The patients/participants provided written informed consent to participate in this study.

## Author contributions

AAk and SF: conceptualization, methodology, experimentation, data analysis, manuscript writing, and review and editing. ZI, MI, and AA helped in experimental data analysis and revision. JKA, TZ and AFA helped in resources provision and review of draft. AAk: final manuscript proof reading. All authors read and approved the final manuscript.

## Funding

The authors are thankful to the researchers supporting project number (RSP2023R335), King Saud University, Riyadh, Saudi Arabia.

## Conflict of interest

The authors declare that the research was conducted in the absence of any commercial or financial relationships that could be construed as a potential conflict of interest.

## Publisher’s note

All claims expressed in this article are solely those of the authors and do not necessarily represent those of their affiliated organizations, or those of the publisher, the editors and the reviewers. Any product that may be evaluated in this article, or claim that may be made by its manufacturer, is not guaranteed or endorsed by the publisher.

## Footnotes

^1^http://www.genomicepidemiology.org/services/

^2^https://www.patricbrc.org/

^3^https://itol.embl.de/
