# Supplementary material for: Whole-genome sequencing of multidrug resistance Salmonella Typhi clinical strains isolated from Balochistan, Pakistan
Source: Front Public Health. 2023 May 16;11:1151805. doi: 10.3389/fpubh.2023.1151805 (PMC10227597; doi:10.3389/fpubh.2023.1151805)
Supplement: Supplementary file 1 [file Data_Sheet_1.zip › Supplementary Material/Supplementary Material Legends.DOCX]

Supplementary Material

**Whole-Genome sequencing of MDR *Salmonella* typhi clinical strains isolated from Balochistan, Pakistan**

**Sareen Fatima, Zaara Ishaq, Muhammad Irfan, Mohammad Y. Alshahrani, Amjad Ali, Ali Akbar^*^**

*** Correspondence:** Corresponding Author: Ali Akbar, Department of Microbiology, University of Baluchistan, Quetta 87300, Balochistan Pakistan

E-mail: [aliakbar.uob@gmail.com](mailto:aliakbar.uob@gmail.com)

# Supplementary Figures and Tables

## Supplementary Figures

**
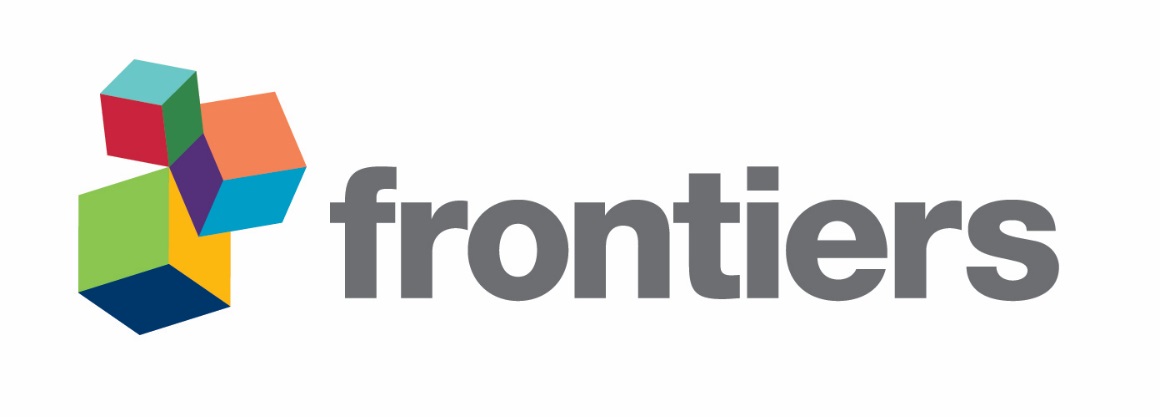
**

**Supplementary Figure 1.** Agarose gel (2%) electrophoresis shows amplification fragments of *invA* (284 bp) and *fliC* (495 bp) genes of *Salmonella typhi* by Duplex PCR: Lanes 01-07 showing the positive amplification of *invA* (284 bp) and *fliC* (495 bp) genes of Salmonella typhi. M1/M2: 100 bp apart DNA marker. +C: positive control. –C: Negative Control.

**Supplementary** **Figure 2** Circular visualization of QS194 isolate showing ORFs, GC Content and GC Skew in **(a)**, Prokka genome annotation in **(b)** antibiotic resistance genes in **(c)** and CRISPR-Cas9 genes and clusters in **(d)**

**Supplementary Figure 3** Circular visualization of QS430 isolate showing ORFs, GC Content and GC Skew in **(a)**, Prokka genome annotation in **(b)** antibiotic resistance genes in **(c)** and CRISPR-Cas9 genes and clusters in **(d)**

**Supplementary Figure 4** Circular visualization of QS468 isolate showing ORFs, GC Content and GC Skew in **(a)**, Prokka genome annotation in **(b)** antibiotic resistance genes in **(c)** and CRISPR-Cas9 genes and clusters in **(d)**

## Supplementary Tables

**Supplementary Table 1** Nucleotide sequence of the primers used for the detection of *Salmonella typhi*.

**Supplementary Table 2** Metadata regarding these genomes including strain name, Biosample, Bioproject, accession numbers, GC% sequence type, isolate type and geographical location etc.

**Supplementary Table 3** Biochemical Testing of *Salmonella typhi*.

**Supplementary Table 4** Antimicrobial Susceptibility of *Salmonella typhi.*
