# Supplementary material for: Whole-genome sequencing of multidrug resistance Salmonella Typhi clinical strains isolated from Balochistan, Pakistan
Source: Front Public Health. 2023 May 16;11:1151805. doi: 10.3389/fpubh.2023.1151805 (PMC10227597; doi:10.3389/fpubh.2023.1151805)
Supplement: Supplementary file 1 [file Data_Sheet_1.zip › Supplementary Material/Table 1.PDF]

Supplementary Table 1 Nucleotide sequence of the primers used for the detection of *Salmonella typhi*

Gene	Primer 5` - 3`	Expected Amplicon size	Target Organism	References
invA	F: GTGAAATTATCGCCACGTTCGGGCAA<br>R: TCATCGCACCGTCAAAGGAACC	284 pb	*Salmonella spp.*	Rahn *et al.*, 1992; (17)
fliC	F- ACTGCTAAAACCACTACT<br>R- TTAACGCAGTAAAGACAG	495 bp	*Salmonella typhi*	Song *et al.*, 1993, (18)
