# Supplementary material for: Whole-genome sequencing of multidrug resistance Salmonella Typhi clinical strains isolated from Balochistan, Pakistan
Source: Front Public Health. 2023 May 16;11:1151805. doi: 10.3389/fpubh.2023.1151805 (PMC10227597; doi:10.3389/fpubh.2023.1151805)
Supplement: Supplementary file 1 [file Data_Sheet_1.zip › Supplementary Material/Table 3.PDF]

Supplementary Table 3 Biochemical Testing of *Salmonella typhi*

Microorganism	TSI	Citrate	Urease	Sulfate	Indole	Motility
Salmonella typhi	Alk/A<br>H_2S +ve (weak)	+	-	+<br>(weak)	-	+
