# Supplementary material for: Whole-genome sequencing of multidrug resistance Salmonella Typhi clinical strains isolated from Balochistan, Pakistan
Source: Front Public Health. 2023 May 16;11:1151805. doi: 10.3389/fpubh.2023.1151805 (PMC10227597; doi:10.3389/fpubh.2023.1151805)
Supplement: Supplementary file 1 [file Data_Sheet_1.zip › Supplementary Material/Table 4.PDF]

Supplementary Table 4 Antimicrobial Susceptibility of *Salmonella typhi*

No.	Antibiotics	Sensitivity
1	Ampicillin (10 µg)	R
2	Chloramphenicol (30 µg)	R
3	Streptomycin (10 µg)	S
4	Sulfonamide (300 µg)	R
5	Trimethoprim (5 µg)	R
6	Ciprofloxacin (5 µg)	R
7	Tetracycline (30 µg)	S
8	Gentamicin (10 µg)	R
9	Nalidixic Acid (30µg)	S
10	Cefotaxime (5 µg)	R
11	Mecillinam (10 µg)	S
12	Imipenem (10 µg)	R
