# Supplementary figures and images for: Whole-genome sequencing of multidrug resistance Salmonella Typhi clinical strains isolated from Balochistan, Pakistan
Source: Front Public Health. 2023 May 16;11:1151805. doi: 10.3389/fpubh.2023.1151805 (PMC10227597; doi:10.3389/fpubh.2023.1151805)

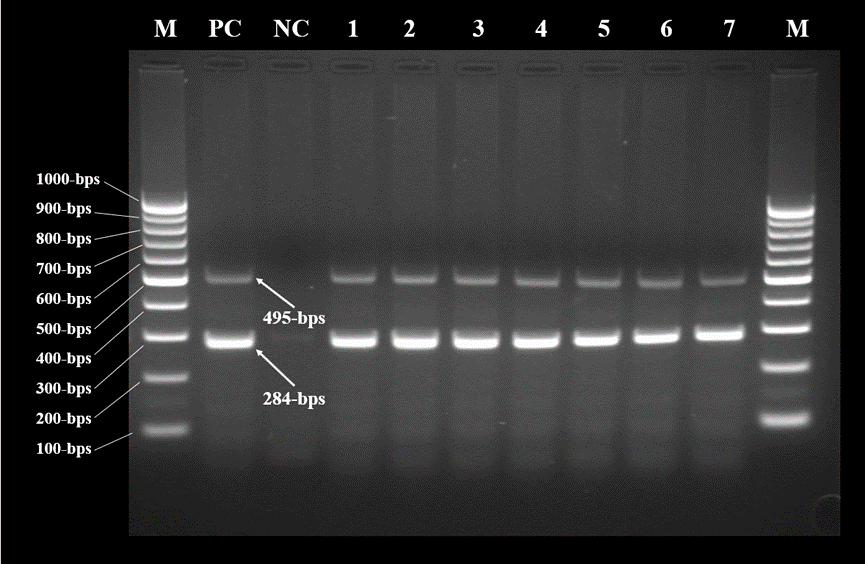

Supplement: Supplementary file 1 [file Data_Sheet_1.zip › Supplementary Material/Figure 1.PNG]
