# Supplementary material for: Whole-genome sequencing of multidrug resistance Salmonella Typhi clinical strains isolated from Balochistan, Pakistan
Source: Front Public Health. 2023 May 16;11:1151805. doi: 10.3389/fpubh.2023.1151805 (PMC10227597; doi:10.3389/fpubh.2023.1151805)
Supplement: Supplementary file 1 [file Figure 2.pdf › Supplementary Material/Figure 2.pdf]

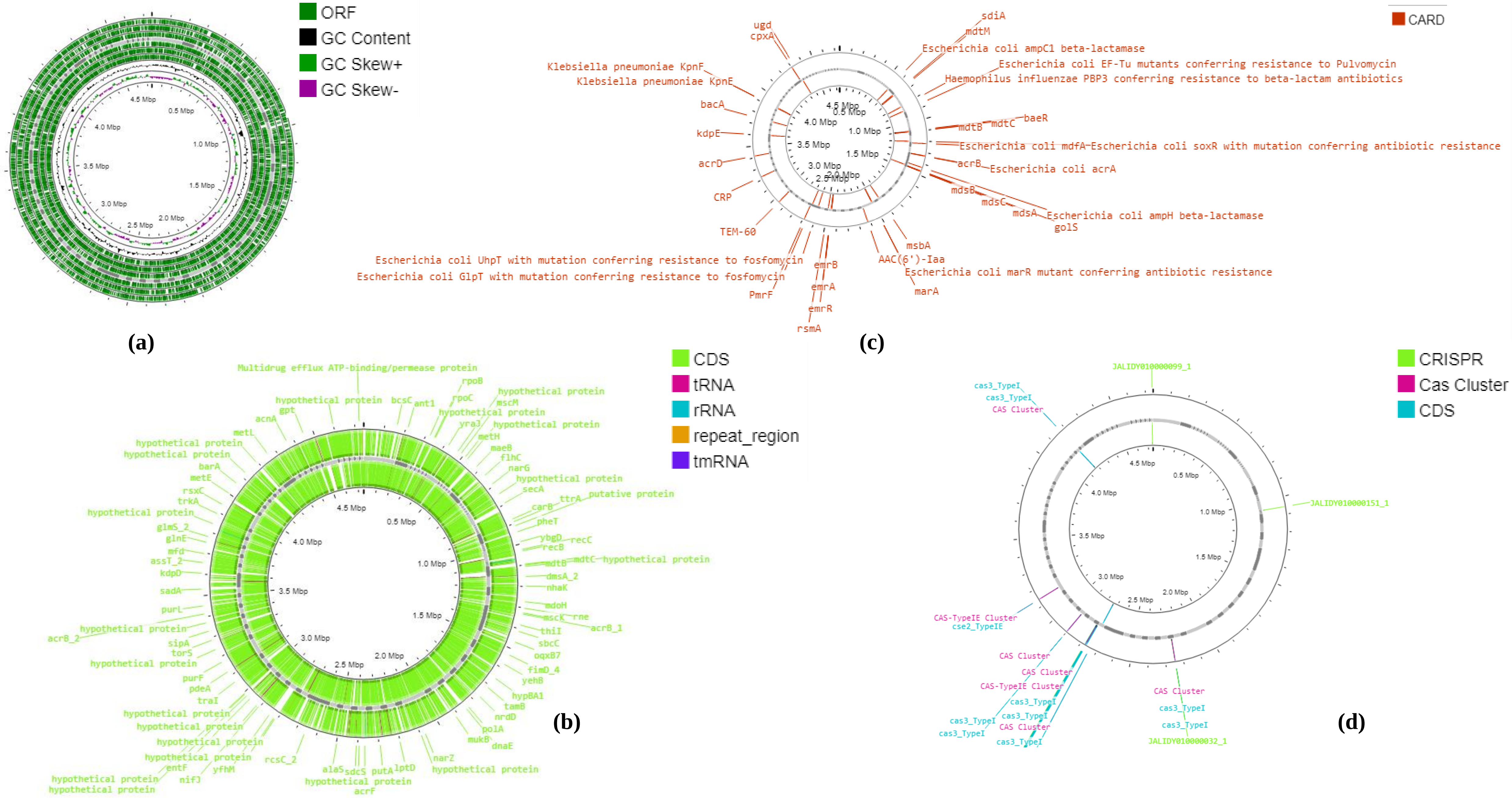

Figure S2 Circular visualization of QS194 isolate showing ORFs, GC Content and GC Skew in **(a)**, Prokka genome annotation in **(b)** antibiotic resistance genes in **(c)** and CRISPR-Cas9 genes and clusters in **(d)**
